# Knowledge, attitude, and practice regarding tuberculosis in a labor-intensive industrial district

**DOI:** 10.3389/fpubh.2024.1431060

**Published:** 2024-11-13

**Authors:** Jin Li, Liping Lu, Jinyan Zou, Yong Li, Lijuan Fu, Qi Zhao

**Affiliations:** ^1^Department of Tuberculosis Control, Songjiang District Center for Disease Control and Prevention, Shanghai, China; ^2^Department of Epidemiology and Biostatistics, School of Public Health, Fudan University, Shanghai, China

**Keywords:** tuberculosis, KAP, labor-intensive, worker, preventive education

## Abstract

**Background:**

In China, tuberculosis (TB) is a major contributor to deaths caused by infectious diseases, with a significant number of cases remaining undetected. Lack of knowledge could heighten the chances of infecting TB. Due to the lack of information on the knowledge, attitudes, and practices (KAP) related to TB among labor-intensive businesses, the study aimed to evaluate the TB KAP within this demographic.

**Methods:**

A descriptive cross-sectional survey was conducted among 1,007 participants from March 1 to 28, 2023. A survey was created for assessing knowledge, attitudes, and practices related to tuberculosis, and was sent to employees within the company. We utilize t-tests, ANOVA, and multiple linear regression to investigate the relationship between TB knowledge, attitudes, and practices and various influencing factors.

**Results:**

The mean good scores rate for TB KAP were 43.5, 23.5, and 75.3%, respectively. Native, female, and workers living in their own houses had a higher score in TB knowledge. Native, non-operators, and workers with a history of TB contact had higher scores in TB practice. Regarding the multivariable linear regression analysis, sex, seniority, birthplace, marital status, and sources of information were associated with greater knowledge; monthly income categories were associated with greater attitude; and position, birthplace, and contact history were associated with greater practice.

**Conclusion:**

The survey results lead to the assumption that the level of KAP toward TB is not high among manufacturing workers in Songjiang district. Therefore, it is crucial to enhance tuberculosis knowledge, attitudes, and practices within this demographic.

## Introduction

After COVID-19, Tuberculosis (TB) remains the second leading cause of death worldwide from a single infectious disease, with approximately 10.6 million new cases and 1.3 million fatalities reported in 2021 (1). Global efforts to care for and prevent TB are being hindered by health service pressures following 2 years of COVID-related disruptions (2). Consequently, access to TB diagnosis and treatment has been severely affected, along with the burden of TB (3). In 2022, China ranked as the third largest nation globally with a significant TB burden, where a considerable number of TB cases went undetected (4).

Since the 1990s, Shanghai has been a highly developed and sought-after destination in China for people moving from rural to urban areas. With a large number of migrants from cities with high incidences of TB to low-incidence areas like Shanghai, challenges for TB control have emerged. Especially in Songjiang district, the first and biggest export industrial zone in Shanghai. By 2021, Songjiang had a population of 1.93 million, with 57.6% being internal migrants, and more than 80,000 migrants congregated in industrial areas (5). Most migrants opt to work in labor-intensive businesses, where the close quarters and frequent interactions in workshops and dorms contribute to the spread of TB (6). Due to busy work, they usually delayed in seeking health care. Thus, once diagnosed with TB, it is easy to result in transmission in workshops and dormitories and even outbreaks.

Worldwide, there has been a significant increase in worldwide reports of individuals newly identified with TB, while notifications in China have been steadily decreasing for over a decade (1). This indicates that there is a significant number of individuals who contracted TB in the past but were not identified, highlighting the need for increased efforts to enhance the detection rate (7). Having a good understanding is essential for developing a strategy to control TB, and filling in knowledge gaps related to TB prevention is crucial for eradicating TB (8). Limited evidence exists on the current KAP regarding TB among migrant workers in Shanghai, despite continued efforts in TB control and prevention. Inadequate knowledge about TB among this high-risk population, made it hard to achieve early case detection and treatment, thereby causing community transmission (9). Hence, estimating their KAP has a major implication in undertaking measures to achieve the TB elimination goals.

Several studies have investigated TB transmission among migrants in the study area and disclosed that Shanghai is facing great challenges from migrants (6, 10–13). Cities with low TB incidence prioritize minimizing the risk of TB importation from high-burden cities and addressing the needs of migrants to achieve TB elimination (14). Thus, it is quite necessary to do more research for a better understanding of KAP related to TB among migrant workers. The study aimed to assess the knowledge, attitudes, and practices regarding tuberculosis in a labor-intensive company in Shanghai, China, to enhance the existing conditions.

## Materials and methods

### Study design and population

The study was conducted in an electronic manufacturing enterprise, which is one of the world’s largest notebook manufacturers, located in southwestern Shanghai. The factory zone takes up over 660,000 square meters, while the living zone takes up 330,000 square meters. The enterprise contains seven production zones, and over 20 dormitory buildings (without private bathrooms), which can accommodate a maximum of over 80 thousand employees. And there are nearly 30 thousand employees in the company over the survey period. A study with a cross-sectional design was carried out between March 1 and 28, 2023. A single population proportion formula was used to estimate the sample size. The following assumptions were made: with the assumption of the proportion of awareness about TB 57% (15), 95% confidence level and a 5% margin of error, and allowance for a non-response rate of 10%, the estimated sample size was 413, but we were able to conduct 1,048 interviews with participants in the study successfully. Before the data was officially collected, we carried out an internal pilot survey involving 76 workers from all factory departments. The final questionnaire was revised according to the pilot survey and opinions from specialists in this field.

Due to the frequent staff rotation, a convenience sampling method was employed to select participants. The questionnaire was delivered both online and offline simultaneously. The survey was conducted using an online questionnaire through “Wenjuanxing” (a professional online questionnaire survey platform). The employees could get the questionnaire by scanning QR codes on the posters put within the workshops, dormitories, and dining halls and on the company’s internal website. Besides, doctors and nurses in the infirmary were asked to help in distributing and retrieving the questionnaire. All employees could participate in the survey and they are required to fill in their employee ID when submitting the questionnaire for verification by our researchers.

### Questionnaire design

The questionnaire for this study purpose was developed based on WHO TB guidelines to develop knowledge, attitude, and practice surveys (16). The questionnaire had four essential parts and consisted of 30 multiple-choice questions. The initial section included fundamental sociodemographic inquiries, including age, sex, marital status, place of birth, employment dates, employee identification number, educational level, housing status, job title, and monthly income average (in yuan), as well as history of previous tuberculosis exposure. The second part focused on the knowledge of participants. Participants were questioned regarding the etiology, symptoms, modes of transmission, preventative measures, vulnerable populations, treatment regimen, and associated costs. The third section focused on exploring perceptions of tuberculosis, including how it is viewed as a health issue in the workplace and community, the severity of the participant’s perception of tuberculosis, and whether they would conceal a tuberculosis diagnosis. The final part measured practice for health-seeking and preventative behavior when they or people around them had TB, such as willingness to seek medical treatment if respondents thought they had TB symptoms, and whether the participant would persuade TB patients to visit a health facility. The questionnaire can be found in the Supplementary material.

### Data sources, measurement, and analysis

Data generated were organized in Microsoft Excel, and statistical analysis was carried out using R software version 3.6.1. Participants were scored on their KAP of TB from 18 selected questions, including 12 single and 8 multiple-choice questions. Answers to the single-choice knowledge questions were scored as “1” for correct and “0” for incorrect for each question. Responses to questions with multiple correct answers were classified as good, fair, or poor based on the accuracy of the selection. To calculate the score, a good answer was scored as “2,” a fair answer as “1” and a poor answer as “0.” Good responses are those that select the correct answer, fair responses select both correct and incorrect answers, and poor responses select only the incorrect answer. For example, the question “How can a person prevent getting TB?” has more than one correct answer. Choosing all correct answers such as covering mouth and nose when coughing or sneezing, wearing masks in public, opening windows frequently, and receiving prophylactic treatment were given a score of “2.” Choosing both incorrect answers such as avoiding sharing dishes and any correct answer was given a score of “1,” while choosing only incorrect answers scored “0.” The outcome variable was generated according to the total score and separate scores, which were calculated by adding values to each question. The scoring criteria can be found in the Supplementary material.

### Statistical analysis

Descriptive data were summarized and presented as counts and percentages (proportions). The knowledge, attitude, and practice scores were calculated individually. The mean scores of different groups were also calculated and compared. Descriptive statistics, Student’s t-tests, or one-way ANOVA were used to analyze differences in different groups. Determinant factors related to knowledge, attitude, and practice were analyzed using a multivariable linear regression analysis. The method of stepwise regression was applied for the model selection based on the criteria of the lowest Akaike Information Criterion (AIC). All measured characteristics were included within this univariable analysis. Variables that showed an association with TB KAP at a significance level of *p* < 0.1 in the univariate analysis were included in the multiple linear regression analysis. *p*-value less than 0.05 was considered statistically significant.

### Ethical approval

The Scientific and Technology Committee of Songjiang District was informed about the research content to get permission. Approval was granted by the Ethical Review Committee at Shanghai Municipal Center for Disease Control and Prevention (No. 2023–28). To protect the confidentiality of the respondents, their personal information was removed and a code number was given to each participant. Informed written consent was obtained from the respondents before they participated in the research.

## Results

### Demographics and characteristics of participants

In total, 1,148 employees participated in the survey, 141 questionnaires were excluded due to duplicate submission or logic errors, and 1,007 valid questionnaires were received. As shown in Table 1, males accounted for 47.3%. The average age of the individuals involved was 33 years with a standard deviation of 6.5 years, with 886 individuals (88.0%) being younger than 40 years old. The majority (80.0%) were migrant workers. Around 35% of participants were operators working on assembly lines. Most (49.2%) participants had bachelor’s or college degrees. Half of the participants lived in the company dorm with six to eight roommates. Also, most staff (75.2%) described themselves as non-smokers. The demographic characteristics of the participants are shown in Table 1.

**Table 1:** Demographic characteristics of the respondents.

Demographic variable	Total (*N* = 1,007) N (%)
Gender	
Male	476 (47.3)
Female	**531 (52.7)**
Birthplace	
Native	202 (20.00)
Migrant	**805 (80.0)**
Position	
Operator	354 (35.2)
Non-operator	**653 (64.8)**
Age group	
18–25 years	143 (14.2)
26–40 years	**743 (73.8)**
≥40 years	121 (12.0)
Educational status	
Junior high school and below	115 (11.4)
Senior high school or Technical School	397 (39.4)
College or University graduate	**495 (49.2)**
Marital status	
Married	**545 (54.1)**
Unmarried	462 (45.9)
Seniority	
<2 years	254 (25.2)
3–10 years	**352 (35.0)**
11–15 years	200 (19.9)
≥16 years	201 (20.0)
Monthly income categories	
<5,000	228 (22.6)
5,000–8,000	340 (33.8)
≥8,000	**439 (43.6)**
Housing situation	
Buying a house	215 (21.4)
Renting house	280 (27.8)
Dormitory	**512 (50.8)**
Contacted someone with TB	
Yes	69 (6.9)
No	**938 (93.1)**
Smoking	
Never	**757 (75.2)**
Used to smoke	77 (7.6)
Yes	173 (17.2)
Night shift	
Yes	337 (33.5)
No	**670 (66.5)**

Most chosen answers are in bold.

### Knowledge, attitude and practice scoring of TB

Table 2 shows the scoring of participants’ KAP about TB. In general, the average proportion of correct responses for tuberculosis knowledge, attitude, and practice were 43.5, 23.5, and 75.3%. In knowledge dimension, the proportion of correct answers for “Is it acceptable to stop medication?” was relatively high at 84.8%, whereas the scoring for “How can TB be transmitted?” and “How can a person prevent getting TB?” were fair for most participants (94.1 and 96.5%). In attitude dimension, the mean score for “How do you feel about TB patients?” was relatively high at 41.9%, whereas the scoring for “In your community, how do people treat TB patients?” was fair at 86.3%. The practical aspect showed that the highest mean score, at 97.6%, was for the question “Are you willing to actively engage in TB education?,” while the lowest mean score, at 40.9%, was for the question “Would you discuss your illness with your colleagues if you had TB?”

**Table 2:** The scoring of TB knowledge, attitude and practice.

Questions	Good N (%)	Fair N (%)	Poor N (%)
Knowledge			
Do you know the cause of TB?	605 (60.1)	–	402 (39.9)
What are the signs and symptoms of TB?	652 (64.7)	262 (26.0)	93 (9.2)
How can TB be transmitted?	47 (4.7)	948 (94.1)	12 (1.2)
How can a person prevent getting TB?	34 (3.4)	972 (96.5)	1 (0.1)
How long was the course of TB treatment?	443 (44.0)	–	564 (56.0)
Is it acceptable to stop medication?	854 (84.8)	–	153 (15.2)
How expensive do you think TB diagnosis and treatment is in this country?	740 (73.5)	–	267 (26.5)
In your opinion, what proportion of the total China population has a TB infection?	128 (12.7)	–	879 (87.3)
Attitude			
In your opinion, how serious a disease is TB?	381 (37.8)	586 (58.2)	40 (4.0)
How serious a problem do you think TB is in company and community?	54 (5.4)	389 (38.6)	564 (56.0)
If you have TB, what do you feel?	296 (29.4)	576 (57.2)	135 (13.4)
How do you feel about TB patients?	422 (41.9)	223 (22.1)	362 (35.9)
In your community, how do people treat TB patients?	32 (3.2)	869 (86.3)	106 (10.5)
Practice			
Would you discuss your illness with your colleagues if you had TB?	412 (40.9)	–	595 (59.1)
Will you advise people to screen TB if they develop TB symptoms?	457 (45.4)	–	550 (54.6)
Will you advise patients to adhere to treatment recommendations if they had TB?	968 (96.1)	–	39 (3.9)
If you had symptoms of TB, would you go to the health facility?	973 (96.6)	–	34 (3.4)
Will you participate in education about TB actively?	983 (97.6)	–	24 (2.4)

### Determinants of TB knowledge, attitude and practice

Table 3 shows the position of operator is associated with lower practice (3.5 vs. 3.9, *p* < 0.001). Native worker is associated with higher knowledge (6.6 vs. 6.3, *p* = 0.025) and practice (3.9 vs. 3.7, *p* < 0.001), respectively. Women are linked to greater knowledge compared to men (6.5 vs. 6.2, *p* < 0.001). Compared with workers who lived in dormitories or rented dwellings, those who had bought a house in Shanghai had higher knowledge (6.5 vs. 6.3, *p* = 0.050). Employees who have had contact with someone with tuberculosis have lower performance ratings compared to those without such a history (3.5 vs. 3.8, *p* < 0.001).

**Table 3:** Mean score of correct answer by TB KAP.

	Knowledge		Attitude		Practice	
	Mean score	*p*-value	Mean score	*p*-value	Mean score	*p*-value
Position						
Operator	6.3	0.324	5.1	0.168	3.5	**<0.001**
Non-operator	6.4		5.3		3.9	
Birthplace						
Native	6.6	**0.025**	5.4	0.081	3.9	**<0.001**
Migrant	6.3		5.2		3.7	
Sex						
Male	6.2	**<0.001**	5.2	0.380	3.8	0.554
Female	6.5		5.3		3.8	
Age						
18–25 years	6.4	0.265	5.2	0.170	3.7	0.226
26–40 years	6.3		5.2		3.8	
≥40 years	6.6		5.5		3.8	
Seniority						
1–2 years	6.2	0.116	5.1	0.131	3.7	0.053
3–10 years	6.4		5.2		3.7	
11–15 years	6.3		5.3		3.8	
≥16 years	6.5		5.3		3.8	
Educational status						
Junior high school and below	6.6	0.826	5.3	0.523	3.7	0.984
Senior high school or Technical School	6.2		5.2		3.8	
College or University graduate	6.4		5.3		3.7	
Home ownership status						
Yes	6.5	**0.050**	5.3	0.356	3.8	0.351
No	6.3		5.2		3.8	
Night shift						
Yes	6.3	0.353	5.2	0.613	3.8	0.636
No	6.4		5.3		3.8	
Monthly income categories						
<5,000	6.4	0.488	5.4	0.233	3.8	0.624
5,000–8,000	6.3		5.1		3.7	
≥8,000	6.4		5.2		3.8	
Marital status						
Married	6.4	0.640	5.3	0.505	3.8	0.108
Unmarried	6.4		5.2		3.7	
Contact history						
Yes	6.5	0.424	5.1	0.369	3.5	**<0.001**
No	6.4		5.2		3.8	

Statistically significant differences are in bold.

The multivariable linear regression analysis (Table 4) identified an association between female, seniority between 3 to 10 years and over 16 years, native, unmarried workers and learned information about TB through over two sources and greater TB knowledge (*β* = 0.3, 95% CI: 0.2, 0.5; *β* = 0.3, 95% CI: 0.0, 0.5; *β* = 0.3, 95% CI: 0.1, 0.6; *β* = −0.2, 95% CI: −0.4, 0.0; *β* = 0.2, 95% CI: 0.2, 0.4; *β* = 0.2, 95% CI: 0.1, 0.4). Workers with monthly income between 5,000 and 8,000, and over 8,000 had more negative attitudes (*β* = −0.4, 95% CI: −0.6, 0.0; *β* = −0.3, 95% CI: −0.5, 0.0). Additionally, individuals who were not operators, native employees, and did not have a history of contact with tuberculosis were anticipated to exhibit positive behaviors (*β* = −0.5, 95% CI: −0.6, −0.4; *β* = −0.1, 95% CI: −0.2, 0.0; *β* = −0.3, 95% CI: −0.5, −0.1).

**Table 4:** Multiple linear regression analysis of determinants of the percentage of correct answers.

	β	S_b_	*t*	*p*	95%CI
Knowledge					
Constant	5.597	0.300	18.646	**<0.001**	(5.008, 6.186)
Sex (female)	0.347	0.082	4.256	**<0.001**	(0.187, 0.506)
Seniority					
3–10 years	0.251	0.106	2.370	**0.018**	(0.043, 0.459)
11–15 years	0.148	0.130	1.140	0.255	(−0.107, 0.403)
16+ years	0.346	0.134	2.587	**0.010**	(0.083, 0.608)
Birthplace (migrants)	−0.215	0.100	−2.159	**0.031**	(−0.411, −0.020)
Marital status (unmarried)	0.203	0.093	2.167	**0.030**	(0.019, 0.386)
Source of information (over two sources)	0.248	0.082	3.017	**0.003**	(0.087, 0.409)
Attitude					
Constant	5.753	0.257	22.373	**<0.001**	(5.248, 6.257)
Seniority					
3–10 years	0.165	0.130	1.270	0.204	(−0.090, 0.421)
11–15 years	0.298	0.154	1.939	0.053	(−0.004, 0.600)
16+ years	0.283	0.157	1.802	0.072	(−0.025, 0.592)
Monthly income categories					(−0.623, −0.093)
5,000–8,000	−0.358	0.135	−2.648	**0.008**	(−0.570, −0.036)
≥8,000	−0.303	0.136	−2.229	**0.026**	(−0.487, 0.002)
Birthplace (migrants)	−0.242	0.125	−1.946	0.052	
Practice					
Constant	5.052	0.170	29.802	**<0.001**	(4.720, 5.385)
Position (operator)	−0.457	0.048	−9.569	**<0.001**	(−0.551, −0.364)
Birthplace (migrants)	−0.126	0.057	−2.219	**0.027**	(−0.238, −0.015)
Marital status (unmarried)	−0.086	0.045	−1.895	0.058	(−0.175, 0.003)
Contact history (yes)	−0.294	0.090	−3.278	**0.001**	(−0.470, −0.118)
Total					
Constant	17.011	0.524	32.458	**<0.001**	(15.982, 18.039)
Sex (female)	0.339	0.138	2.465	**0.014**	(0.069, 0.609)
Seniority					
3–10 years	0.415	0.178	2.332	**0.020**	(0.066, 0.764)
11–15 years	0.422	0.211	2.005	**0.045**	(0.009, 0.836)
16+ years	0.662	0.216	3.071	**0.002**	(0.239, 1.085)
Monthly income categories					
5,000–8,000	−0.572	0.185	−3.087	**0.003**	(−0.936, −0.208)
≥8,000	−0.365	0.186	−1.960	**0.050**	(−0.730, 0.001)
Position (operator)	−0.654	0.144	−4.551	**<0.001**	(−0.936–0.372)
Birthplace (migrants)	−0.544	0.172	−3.158	**0.002**	(−0.881, −0.206)
Contact history (yes)	−0.498	0.270	−1.841	0.066	(−1.028, 0.033)
Source of information (over two sources)	0.378	0.140	2.697	**0.007**	(0.103, 0.653)

Statistically significant differences are in bold.

### Source of information

**Figure 1:**
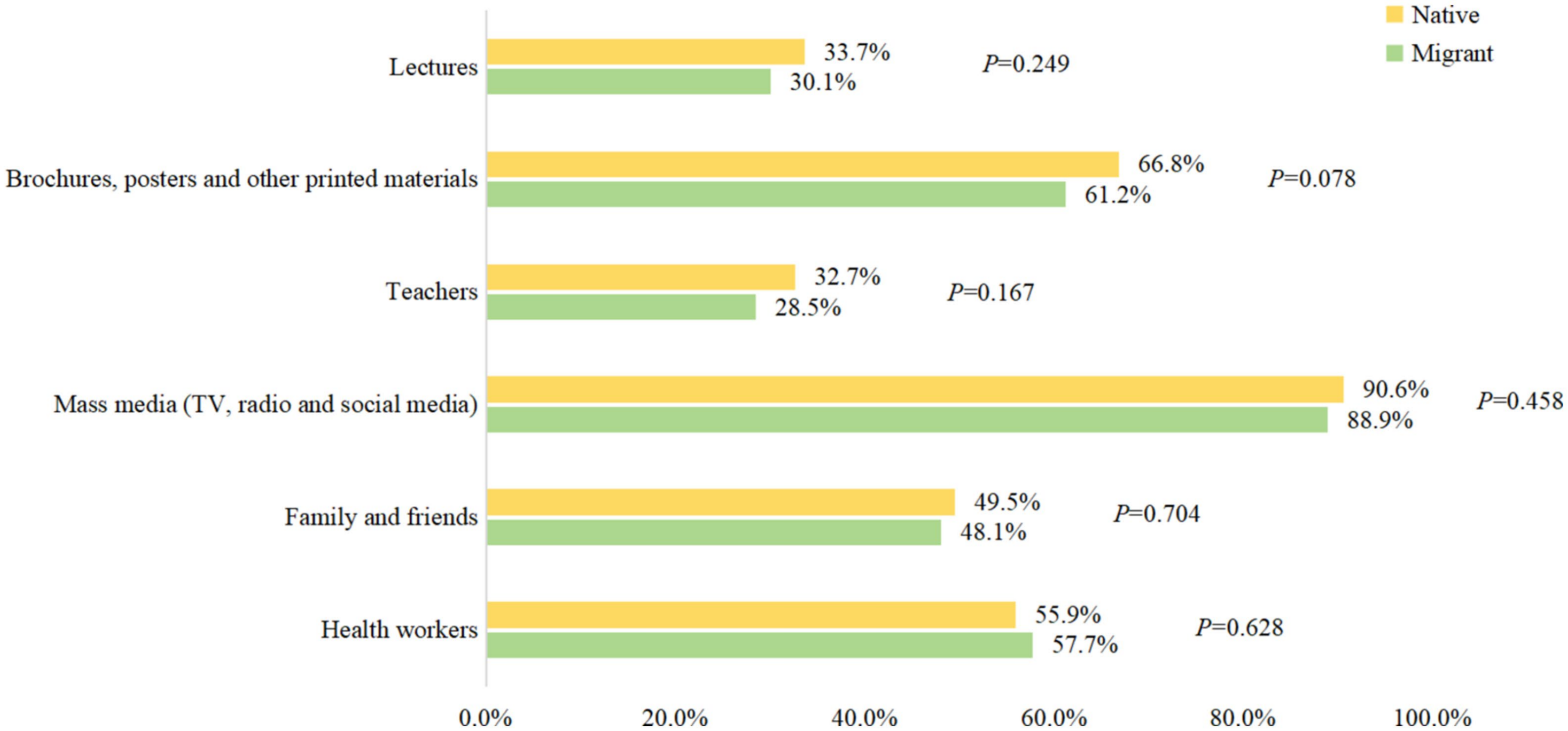
Sources of information on TB that respondents heard about TB.

Figure 1 shows the source of information where respondents heard about TB, while 89% of the respondents heard about TB through mass media, printed materials (61.2%) and health workers (57.7%) were also common sources of information. Teachers only account for 28.5%. Native There is no statistical difference between native and migrant workers.

## Discussion

Our research revealed that employees in labor-intensive businesses generally had a low level of knowledge, attitudes, and practices regarding tuberculosis. Over 50% of the inquiries about TB information were inaccurate, aligning with a similar study done with medical students and another study in Northwest Ethiopia (17, 18). However, the overall KAP level in our study was lower than other surveys conducted in Indonesia (19), Afghanistan (20) and the national average (21), which suggested that there is still a lot of room for improvement.

In our study, although most participants knew the basic knowledge of TB, there were still many misunderstandings. For example, most people only knew the classic symptoms of TB, it is easy to fail to realize TB patients without obvious respiratory symptoms. Although they knew TB is a respiratory tract contagion, they still thought sharing cutlery could transmit the disease. Moreover, quite a few people wrongly thought that TB could not be cured. As for the cost of treatment, many people lack knowledge of Shanghai’s policy about TB treatment. This mistaken perception above would lead to delayed or interruption of treatment. It suggests that we need to spread much more detailed and extensive information about TB prevention, diagnosis and treatment to this population.

As for the attitude and practice in this study, more than half of the participants thought there was no risk of TB in their workplace. With this thought, they may not pay attention to preventing TB in daily work. The level of stigmatization was very high in our study participants, over 90% of participants had negative attitudes about TB. TB stigma has been proven to delay in seeking medical care and reduce treatment compliance (22). Thus, it was not surprising that over 40% of participants chose to conceal the ill of TB to avoid being isolated or even being fired. If they do not take medicine regularly and conceal ill to work, it could easily cause TB outbreak in such densely-populated places.

We find that natives had better KAP scores than migrants. This was also observed by studies in other countries (18, 23). The variance may be ascribed to a deficient health education system in the economically disadvantaged region compared with Shanghai. In addition, the position was associated with practice and total score. It means we need to pay more attention to the front-line operators. Given the long hours and high-intensity work, this group had limited opportunity to obtain TB information. We could explore the strategies to enhance health promotion in the workshop through TV and radio. There was no association between TB knowledge and education status in this study, however, some other studies have reported an association (24, 25). It could be due to that with the admission expansion of colleges in China, the educational level has been rising generally, but the school health education curriculum was seriously lagging.

Although KAP surveys have been conducted among various populations around the world (24–28), to our knowledge, this is the first study that focuses on a labor-intensive enterprise and includes many rural-to-urban migrants. In recent years, although much has been done to popularize TB knowledge like distributing pamphlets or posters, more accurate interventions should be applied to remove TB misconceptions and reduce stigma for different populations. For these particular individuals, who are young, better educated and living overcrowded, we should take some new and engaging measures to improve the effect of TB health education. For example, with the rapid popularization of cell phones, social media has become a new desirable way of getting health information compared to traditional sources (29). Future health interventions should leverage the role of social media to enhance public awareness, meanwhile regulation also needs to be strengthened to prevent the spread of wrong messages. Furthermore, given information from teachers accounted for only 28.5%, the local health department should strengthen TB training of teachers to improve the frequency and coverage of health education.

This study has some limitations. First, given the feature of a cross-sectional survey, it is not possible to determine temporality or causality. Secondly, the questionnaire was delivered online, so the actual response rate cannot be calculated. In addition, the survey was conducted only in one company, so the results cannot be generalized to the whole area. Therefore, further studies need to be done among migrants of other occupations to provide more comprehensive insight into this.

The results of our study could be used by local government to adopt a series of intervention measures to improve the knowledge about TB, thus decreasing the burden of TB in labor-intensive factories. Given the low KAP level of the participants, active TB screening including chest X-rays should be conducted every year to detect patients, especially migrant workers. In addition, the administration of the public health department should strengthen training for TB among healthcare workers in the community or in the company. More company-based TB education should be carried out, especially for migrant workers and front-line operators. Implementing multi-ways health education may enhance the education effect. For example, knowledge of TB should be added to induction training. Meanwhile, electronic screens and websites should be used to show videos about TB knowledge regularly.

## Conclusion

In conclusion, despite all efforts by health facilities, there was still remarkable TB stigma and knowledge gaps on the prevention and treatment of TB. The findings of our study can be used as baseline data for future research to evaluate the effect of health promotion activities. According to the study results, there is still a lot of work on TB educational programs to do for the working population, especially for migrant workers.

## Data Availability

The datasets presented in this article are not readily available because due to the nature of this research, participants of this study did not agree for their data to be shared publicly, so supporting data is not available. Requests to access the datasets should be directed to 466063049@qq.com.
