# Supplementary material for: Knowledge, attitude, and practice regarding tuberculosis in a labor-intensive industrial district
Source: Front Public Health. 2024 Nov 13;12:1431060. doi: 10.3389/fpubh.2024.1431060 (PMC11599202; doi:10.3389/fpubh.2024.1431060)
Supplement: Supplementary file 1 [file Data_Sheet_1.PDF]

You are invited to participant this study which aims to measure the knowledge, attitude and practice regarding tuberculosis in a labor-intensive industrial district. This study is conducted by a research team from Songjiang District Center for Disease Control and Prevention. Your participation is voluntary and all the information you share will be kept completely confidential.

Your employee ID:__________

1.Gender*(Check one)*

☐ Male ☐Female

2.What's your birthplace?*(Check one)*

☐ Shanghai ☐Other province

3.What's your position?*(Check one)*

☐ Operator ☐Non-operator

4.What's your age group?*(Check one)*

☐18-25 years ☐26-40 years ☐⩾40 years

5. What's your education status?*(Check one)*

☐Junior high school and below ☐Senior high school or Technical School

☐College or University graduate

6. What's your marital status? *(Check one)*

☐Married ☐Unmarried

7. How many years have you been in the company? *(Check one)*

☐<2 years ☐3-10 years ☐11-15 years ☐⩾16 years

8. What's your monthly income categories? *(Check one)*

☐<5000 yuan ☐5000-8000 yuan ☐⩾8000 yuan

9. Where do you live? *(Check one)*

☐Dormitory ☐Rented room ☐House that bought by your own

10. Have you contacted someone with TB? *(Check one)*

☐Yes ☐No

11. Do you smoke? *(Check one)*

☐Yes ☐No ☐Used to smoke

12. Do you need to work nights? *(Check one)*

☐Yes ☐No

13. Do you know the cause of TB (tuberculosis)? *(Check one)*

☐Bacteria ☐Virus ☐Other causes

14. What are the signs and symptoms of TB? *(Please check all that are mentioned)*

☐Cough and sputum ☐Hemoptysis ☐Fever ☐Loss of appetite ☐Fatigue

☐Weight loss ☐Night sweats ☐Chest pain

15. How can TB be transmitted? *(Please check all that are mentioned)*

☐Through the air when a person with TB coughs or sneezes

☐By sharing dishes, plates, cups and spoons

☐Through physical contact

☐Mother-to-child

☐By sexual behavior

16. How can a person prevent getting TB? *(Please check all that are mentioned)*

☐Avoid physical contact

☐Covering mouth and nose when coughing or sneezing

☐Avoid sharing dishes

☐Wear masks in public

☐Opening windows frequently

☐Prophylactic treatment

17. How long was the course of TB treatment? *(Check one)*

☐＜6 months ☐6-12 months ☐13-24 months ☐More than 24 months

☐cannot be cured

18. Is it acceptable to stop medication? *(Check one)*

☐Yes ☐No

19. How expensive do you think TB diagnosis and treatment is in this country? *(Check one)*

☐It is free of charge ☐It is reasonably priced ☐It is very expensive

20. In your opinion, what proportion of the total China population has a TB infection? *(Check one)*

☐<5% ☐5%-20% ☐20%-30% ☐＞30%

21. In your opinion, how serious a disease is TB? *(Check one)*

☐Very serious ☐Serious ☐Somewhat serious ☐Not very serious

22. How serious a problem do you think TB is in your country/region? *(Check one)*

☐Very serious ☐Serious ☐Somewhat serious ☐Not very serious

23. If you have TB, what would you feel? *(Please check all that are mentioned)*

☐Sad ☐Embarrassed ☐Scared ☐Amazed ☐Ashamed

☐I have no particular feeling

24. How do you feel about TB patients? *(Read the following choices and check one answer)*

☐I sympathize with them and I try to help

☐I sympathize with them but I tend to stay away from them

☐I am afraid because they may infect me

☐I have no particular feeling

25. In your community, how do people treat TB patients? *(Read the following choices and check one answer)*

☐I sympathize with them and I try to help

☐I sympathize with them but I tend to stay away from them

☐I am afraid because they may infect me

☐I have no particular feeling

26. Would you discuss your illness with your colleagues if you had TB? *(Check one)*

☐Yes ☐No

27. Will you advise people to screen TB if they develop TB symptoms? *(Check one)*

☐Yes ☐No

28. Will you advise patients to adhere to treatment recommendations if they had TB? *(Check one)*

☐Yes ☐No

29. If you had symptoms of TB, would you go to the health facility? *(Check one)*

☐Yes ☐No

30. Will you participate in education about TB actively? *(Check one)*

☐Yes ☐No
