# Supplementary material for: Knowledge, attitude, and practice regarding tuberculosis in a labor-intensive industrial district
Source: Front Public Health. 2024 Nov 13;12:1431060. doi: 10.3389/fpubh.2024.1431060 (PMC11599202; doi:10.3389/fpubh.2024.1431060)
Supplement: Supplementary file 2 [file Data_Sheet_2.PDF]

Knowledge Questions	Answers	Score<br>G= good, F= fair, P = poor	Remarks
**Do you know the cause of TB (tuberculosis)?**	Bacteria<br>Virus<br>Other causes	G<br>P<br>P	
**What are the signs and symptoms of TB?** (Please check all that are mentioned)	Cough and sputum<br>Hemoptysis<br>Fever<br>Loss of appetite<br>Fatigue<br>Weight loss<br>Night sweats<br>Chest pain	1<br>1<br>1<br>1<br>1<br>1<br>1<br>1	Calculation of final score:<br>Good = 6-8<br>Fair = 3-5<br>Poor = 1-2
**How can TB be transmitted?** (Please check all that are mentioned)	Through the air when a person with TB coughs or sneezes<br>By sharing dishes, plates, cups and spoons<br>Through physical contact<br>Mother-to-child<br>By sexual behavior	G<br>P<br>P<br>P<br>P	good= "selecting the accurate answer"<br>poor= "selecting inaccurate answer"<br>fair= "selecting any of inaccurate answers with the accurate answer"
**How can a person prevent getting TB?** (Please check all that are mentioned)	Avoid physical contact<br>Covering mouth and nose when coughing or sneezing<br>Avoid sharing dishes	P<br>G<br>P	good= "selecting the accurate answer"<br>poor= "selecting inaccurate answer"

	Wear masks in public<br>Opening windows frequently<br>Prophylactic treatment	G<br>G<br>G	fair= "selecting any of inaccurate answers with the accurate answer"
**How long was the course of TB treatment?**	＜6 months<br>6-12 months<br>13-24 months<br>More than 24 months<br>cannot be cured	P<br>G<br>P<br>P<br>P	
**Is it acceptable to stop medication?**	Yes<br>No	G<br>P	
**How expensive do you think TB diagnosis and treatment is in this country?**	It is free of charge<br>It is reasonably priced<br>It is very expensive	P<br>G<br>P	
**In your opinion, what proportion of the total China population has a TB infection?**	<5%<br>5%-20%<br>20%-30%<br>＞30%	P<br>P<br>G<br>P	

**Attitude questions**	**Answers**	**Score**	**Remarks**

		G= good, F= fair, P = poor	
**In your opinion, how serious a disease is TB?** (Check one)	Very serious<br>Serious<br>Somewhat serious<br>Not very serious	G<br>F<br>F<br>P	Scoring based on literature about TB
**How serious a problem do you think TB is in your country/region?** (Check one)	Very serious<br>Serious<br>Somewhat serious<br>Not very serious	F<br>G<br>F<br>P	Scoring based on the fact the company is located in one of the highest incident rate areas in Songjiang
**If you have TB, what would you feel?**(Please check all that are mentioned)	Sad<br>Embarrassed<br>Scared<br>Amazed<br>Ashamed<br>I have no particular feeling	1<br>1<br>1<br>1<br>1<br>0	Calculation of final score:<br>Good = 0-1<br>Fair = 2-3<br>Poor = 4-5
**How do you feel about TB patients?** (Read the following choices and check one answer)	I sympathize with them and I try to help<br>I sympathize with them but I tend to stay away from them<br>I am afraid because they may infect me<br>I have no particular feeling	G<br>F<br>P<br>F	good= "selecting the favorable answer"<br>poor= "selecting unfavorable answer"<br>fair= "selecting neutral answer"
**In your community, how do people treat TB**	The community mostly supports and helps him or he	G	good= "selecting the favorable answer"

**patients?**	Most people are friendly, but they generally try to avoid him or her<br><br>Most people not sure they would help him or her<br><br>Most people reject him or her<br><br>Most people have no particular feeling	F<br><br>P<br><br>P<br><br>F	poor= "selecting unfavorable answer"<br><br>fair= "selecting neutral answer"

**Practice questions**	**Answers**	**Score G= good, P = poor**	**Remarks**
**Would you discuss your illness with your colleagues if you had TB?**	Yes<br>No	G<br>P	
**Will you advise people to screen TB if they develop TB symptoms?**	Yes<br>No	G<br>P	
**Will you advise patients to adhere to treatment recommendations if they had TB?**	Yes<br>No	G<br>P	
**If you had symptoms of TB, would you go to the health facility?**	Yes<br>No	G<br>P	
**Will you open the window every day for ventilation?**	Yes<br>No	G<br>P	
